# HTLV‐1 cell‐free DNA in plasma as a potential biomarker in HTLV‐1 carriers and adult T‐cell leukemia‐lymphoma

**DOI:** 10.1002/jha2.725

**Published:** 2023-05-26

**Authors:** Hiroo Katsuya, Hideaki Nakamura, Aya Maeda, Keitaro Ishii, Toshiaki Nagaie, Haruhiko Sano, Haruna Sano, Hidekazu Itamura, Sho Okamoto, Toshihiko Ando, Toshiki Watanabe, Kaoru Uchimaru, Yorifumi Satou, Eisaburo Sueoka, Shinya Kimura

**Affiliations:** ^1^ Division of Hematology Respiratory Medicine and Oncology Department of Internal Medicine Faculty of Medicine Saga University Saga Japan; ^2^ Department of Transfusion Medicine Saga University Hospital Saga Japan; ^3^ Department of Practical Management of Medical Information Graduate School of Medicine St. Marianna University Kawasaki Japan; ^4^ Laboratories of Tumor Cell Biology Department of Computational Biology and Medical Sciences Graduate School of Frontier Sciences The University of Tokyo Tokyo Japan; ^5^ Division of Genomics and Transcriptomics Joint Research Center for Human Retrovirus Infection Kumamoto University Kumamoto Japan; ^6^ Department of Clinical Laboratory Medicine Faculty of Medicine Saga University Saga Japan

**Keywords:** ATL, HTLV‐I, lymphoid malignancies, quantitative PCR

## Abstract

Viral cell‐free DNA (cfDNA) in plasma has been widely evaluated for detecting cancer and monitoring disease in virus‐associated tumors. We investigated whether the amount of cfDNA of human T‐cell leukemia virus type 1 (HTLV‐1) correlates with disease state in adult T‐cell leukemia‐lymphoma (ATL). HTLV‐1 cfDNA in aggressive ATL was significantly higher than that in indolent ATL and asymptomatic carriers. Notably, patients with lymphoma type represented higher HTLV‐1 cfDNA amount than chronic and smoldering subtypes, though they had no abnormal lymphocytes in the peripheral blood. HTLV‐1 cfDNA can be a universal biomarker that reflects the expansion of ATL clones.

1

Human T‐cell leukemia virus type 1 (HTLV‐1) is integrated into the human genomic DNA and causes persistent and usually asymptomatic infections. However, some individuals experience a lymphoid malignancy of infected CD4^+^ T cells, known as adult T‐cell leukemia‐lymphoma (ATL). ATL is classified into four clinical subtypes: acute, lymphoma, chronic, and smoldering types, based on clinical characteristics and the natural history of the disease [1]. Chronic type is further divided into two categories according to the presence of unfavorable prognostic factors, defined by a high level of blood urea nitrogen, a high level of lactate dehydrogenase (LDH), or a low albumin level [2]. Patients with acute, lymphoma, and unfavorable chronic types rapidly progress requiring quick initiation of chemotherapy [3]. In contrast, patients with favorable chronic and smoldering type generally progress slowly and are therefore recommended to be carefully monitored by watchful waiting if they are asymptomatic. However, it has been known that even patients with the same subtypes can present with various clinical courses and survival times. Other than clinical subtypes, previous studies have reported the potential usage of several predictive and prognostic biomarkers for ATL in HTLV‐1‐infected individuals, such as soluble interleukin‐2 receptor (sIL‐2R), serum albumin [4, 5], C‐reactive protein, corrected serum calcium [6, 7], the downregulation of CD7 expression in CD4^+^ and cell adhesion molecule 1 (CADM1)^+^ cells [8, 9], and HTLV‐1 proviral load (PVL) in peripheral blood mononuclear cells (PBMCs) [10]. Also, ATL can be detected early through quantification of T‐cell receptor Vβ subunit on T cells infected with HTLV‐1 using flow cytometry and detection of largely expanded HTLV‐1 clones using next‐generation sequencing [11, 12]. High HTLV‐1 PVL, defined as more than four copies per 100 PBMCs, has been reported as one of the major risk factors for ATL development in HTLV‐1 carriers. On the other hand, HTLV‐1 PVL is not a prognostic factor for individuals with ATL, because PVL is high even in patients with indolent ATL.

Cell‐free viral DNA in plasma has been widely evaluated as a liquid biopsy for detecting cancer and monitoring disease in patients with virus‐associated tumors. For instance, human papillomavirus (HPV) cell‐free DNA (cfDNA) in plasma is detectable and a good biomarker for tumor burden and presence of metastases in patients with HPV‐associated oropharyngeal or cervical cancer [13, 14]. Epstein–Barr virus (EBV) cfDNA in plasma is also a good indicator for response and overall survival in patients with EBV‐associated lymphoma [15]. The association between HTLV‐1 cfDNA amount in plasma and disease states in HTLV‐1‐infected individuals has not yet been fully clarified. Here, we assessed the potential of HTLV‐1 cfDNA as a desirable biomarker for HTLV‐1‐infected individuals using quantitative PCR.

We enrolled 67 previously untreated patients: 42 asymptomatic carriers, nine smoldering types, seven chronic types, five lymphoma types, and four acute types (Supporting Information). All patients gave written informed consent in accordance with the Declaration of Helsinki. This study was approved by the existing institutional review board at Saga University. Plasma was spun down from whole‐blood samples with an EDTA 2Na tube (3000 rpm for 20 min) within 4 h after blood collection, and cfDNA was extracted from 1 mL plasma using Maxwell RSC. Genomic DNA was extracted from PBMCs using the DNeasy kit (QIAGEN) for HTLV‐1 PVL measurement. Droplet digital PCR (ddPCR) was performed using primers and a probe targeting a conserved region in HTLV‐1 pX region as previously described [16]. PCR cycles were performed in a QX200 Droplet Digital PCR system (Bio‐Rad) with the following settings: 95°C for 10 min, followed by 39 cycles of 94°C for 30 s, 58°C for 60 s, and final 98°C for 10 min and 4°C for hold. Data were analyzed using QuantaSoft software (Bio‐Rad). Statistical significance was analyzed by Prism 9 software (version 9.4.1; GraphPad Software).

We first evaluated the amount of HTLV‐1 cfDNA according to clinical subtypes (Figure 1A). The median values of HTLV‐1 cfDNA were 625 copies/mL (range: 68.8–1550), 1650 copies/mL (range: 837.5–3787.5), 56.3 copies/mL (range: 28.8–1213), 17.5 copies/mL (range: 0–287.5), and 0.7 copies/mL (range: 0–33.8) in patients with acute, lymphoma, chronic, smoldering, and asymptomatic carriers, respectively. Patients with lymphoma type represented the highest HTLV‐1 cfDNA amount in all clinical subtypes, though they had low values of HTLV‐1 PVL in PBMCs. The cfDNA levels of lymphoma type were significantly higher than those of chronic, smoldering types, and asymptomatic carriers (lymphoma vs. chronic, *p* = 0.01; lymphoma vs. smoldering, *p* = 0.001; lymphoma vs. asymptomatic carriers, *p* < 0.0001). The cfDNA levels of lymphoma type also tended to be higher than that of acute type, though there was no significant difference (median, 625 and 1650 copies/mL in acute and lymphoma types, respectively, *p* = 0.111). The HTLV‐1 cfDNA levels of acute type were significantly higher than those of smoldering type and asymptomatic carries (acute vs. smoldering, *p* = 0.032; acute vs. asymptomatic carriers, *p* < 0.0001), but were not significantly different from those of chronic type (*p* = 0.164). However, the two patients with chronic type having the highest HTLV‐1 cfDNA amount (shown in circles in Figure 1A) were classified as unfavorable chronic type and high‐risk indolent ATL according to the Shimoyama classification and indolent ATL prognostic index, respectively, and these patients were recommended for immediate initiation of chemotherapy. By contrast, the median PVLs were 69.3% (range: 9.1%–88.5%), 8.5% (range: 1.2%–14.8%), 76.0% (range: 14.5%–212.4%), 16.3% (range: 0.0%–60.1%), and 6.0% (range: 0.1%–20.9%) in patients with acute, lymphoma, chronic, smoldering, and asymptomatic carriers, respectively (Figure 1B). There were no differences in PVL between acute and chronic (*p* = 0.788), and acute and smoldering types (*p* = 0.199). These findings show that HTLV‐1 cfDNA is a more promising biomarker to evaluate disease states in HTLV‐1‐infected individuals compared to HTLV‐1 PVL of PBMCs.

**FIGURE 1:**
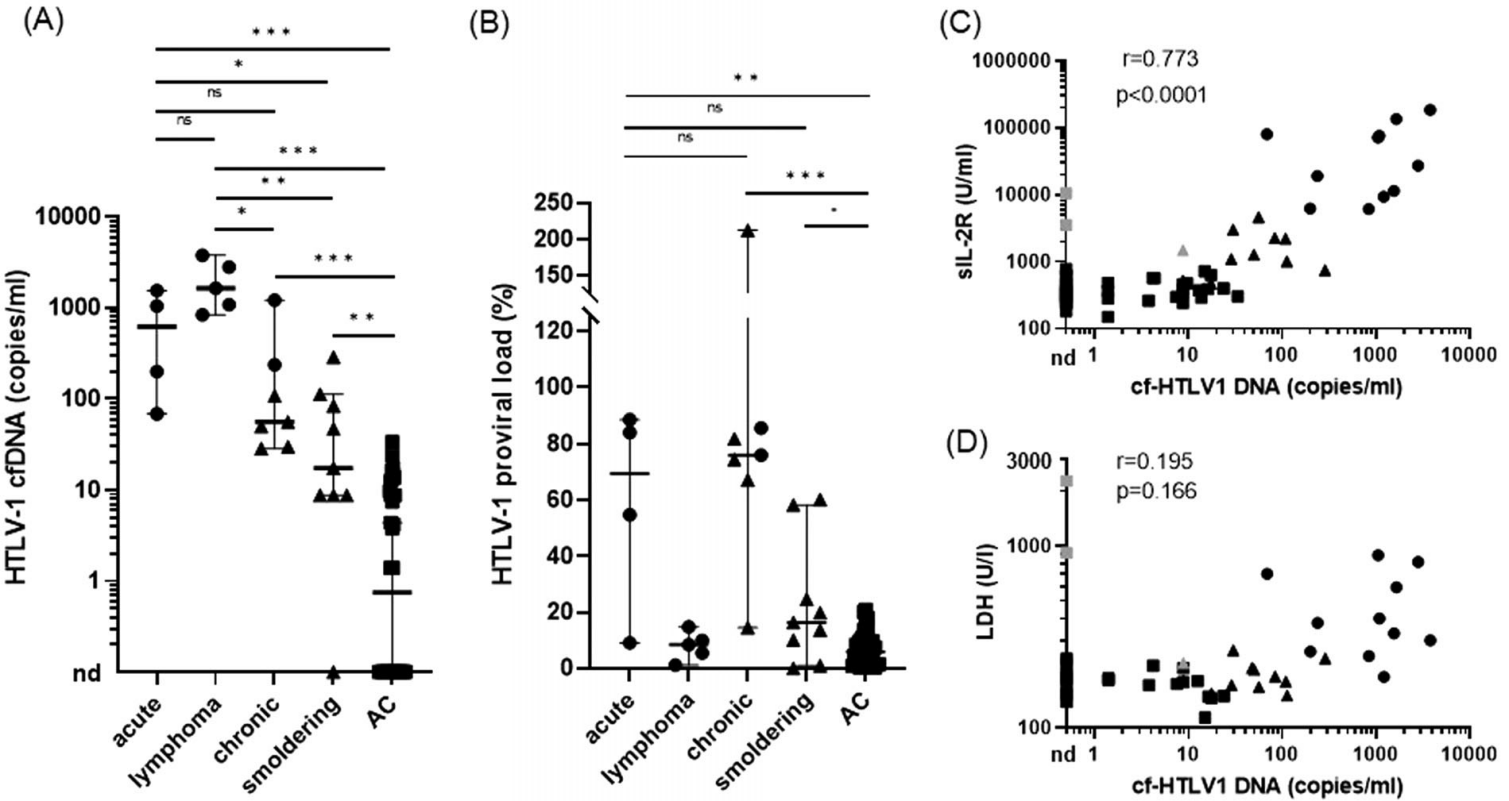
Measurement of HTLV‐1 cell‐free DNA (cfDNA) in plasma and proviral loads in peripheral blood mononuclear cells (PBMCs). (A) HTLV‐1 cfDNA copies per milliliter of plasma were quantified using droplet digital PCR (ddPCR) in patients with four acute types, five lymphoma types, seven chronic types (two unfavorable and five favorable chronic types), nine smoldering types, and 42 asymptomatic carriers. HTLV‐1 cfDNA were not detected in one patient with smoldering type and 19 with asymptomatic carriers. (B) HTLV‐1 proviral loads in PBMCs were quantified using ddPCR in the same patients. (C) Correlation between HTLV‐1 cfDNA and soluble interleukin‐2 receptor (sIL‐2R). A significant correlation between sIL‐2R and HTLV‐1 cfDNA levels was observed. (D) Correlation between HTLV‐1 cfDNA and serum LDH. HTLV‐1 cfDNA level was not correlated with LDH. The value of *r* indicates correlation coefficient. Each circle, triangle, and square represent an individual with aggressive ATL (acute, lymphoma, unfavorable chronic types), indolent ATL (favorable chronic and smoldering types), and asymptomatic carrier, respectively. The gray marks in (C) and (D) indicate the three individuals in Table 1. The bars indicate median values with 95% confidence interval. Statistical significance was obtained by Mann–Whitney *U* test. *p < 0.05, **p < 0.005, ***p < 0.0001. HTLV‐1, human T‐cell leukemia virus type 1; ns, not significant; nd, not detected.

Next, we assessed the correlation between HTLV‐1 cfDNA and other clinical factors. HTLV‐1 cfDNA was well correlated with sIL‐2R (*r* = 0.773, *p* < 0.0001, Figure 1C), but not with serum LDH (*r* = 0.195, *p* = 0.166, Figure 1D), PVL (*r* = 0.13, *p* = 0.31, data not shown), and the populations of CADM1^+^ CD7^–^ in CD4^+^ cells (*r* = 0.17, *p* = 0.20). In contrast, HTLV‐1 PVL of PBMCs was correlated with the populations of CADM1^+^ CD7^–^ in CD4^+^ cells (*r* = 0.81, *p* < 0.0001), but not with sIL‐2R (*r* = 0.04, *p* = 0.77) and LDH (*r* = 0.05, *p* = 0.73). HTLV‐1 cfDNA was highly correlated with sIL‐2R, which reflects the overall tumor burden in ATL patients. The soluble form of IL‐2R is generated by the proteolytic cleavage of membrane IL‐2Rα from activated T cells [17]. The sIL‐2R value is, therefore, influenced by not only the disease states of ATL but also infection and inflammatory disorders. In fact, we experienced two HTLV‐1‐infected carriers who developed diffuse large B‐cell lymphoma presented with high values of sIL‐2R (10,514 and 3545 U/mL), while HTLV‐1 cfDNA was not detected in their serum (Table 1). Additionally, another patient with smoldering type ATL showed right axillary lymphadenopathy and an elevated sIL‐2 level (1501 U/mL), and therefore, we suspected a transformation to aggressive ATL. However, histological analysis of the swollen lymph node revealed reactive lymphadenopathy, which is consistent with the low levels of HTLV‐1 cfDNA in the patient (8.8 copies/mL). These findings suggested that the amount of HTLV‐1 cfDNA has a higher specificity for ATL diagnosis compared to that of sIL‐2R.

**TABLE 1:** Clinical information of two HTLV‐1 carriers who developed DLBCL and a patient with smoldering ATL who was diagnosed with reactive lymphadenopathy.

Patient	Age	Clinical subtype	Histopathological diagnosis	HTLV‐1 cell‐free DNA (copies/mL)	sIL‐2R (U/mL)	LDH (U/L)
1	78	Asymptomatic carrier	DLBCL	0.0	10,514	2273
2	82	Asymptomatic carrier	DLBCL	0.0	3545	920
3	73	Smoldering	Reactive lymphadenopathy	8.8	1501	216

*Note*: The normal value ranges of sIL‐2R and LDH are 122–496 (U/mL) and 124–222 (U/L), respectively.

Abbreviations: DLBCL, diffuse large B‐cell lymphoma; LDH, lactate dehydrogenase; sIL‐2R, soluble interleukin‐2 receptor.

HTLV‐1 cfDNA is a potential biomarker in HTLV‐1‐infected individuals from asymptomatic carriers to aggressive ATL. We demonstrated that HTLV‐1 cfDNA levels in patients with aggressive ATL were significantly higher than those in indolent ATL and asymptomatic carriers, indicating that HTLV‐1 cfDNA reflects the overall tumor burden and disease progression in HTLV‐1 individuals. HTLV‐1 PVL has been reported to be a predictive marker of ATL development from asymptomatic carriers. However, the PVL values do not correspond well with the overall tumor burden and the disease states as shown in Figure 1B. The population of CADM1^+^CD7^dim^ cells evaluated by flow cytometric analysis increases in patients with ATL development (from asymptomatic carriers to indolent ATL) [9], while the population of CADM1^+^CD7^–^ cells expands in patients who progress from indolent to aggressive ATL [8]. However, this flow cytometric analysis is not useful for predicting the development of lymphoma‐type ATL because of the lack of expansion of abnormal cells in PBMCs. Early detection of lymphoma‐type ATL using PBMCs is also difficult even by quantification of T‐cell receptor Vβ and the evaluation of clonality through next‐generation sequencing. We demonstrated that the amount of HTLV‐1 cfDNA was extremely high even in patients with lymphoma type (Figure 1A), suggesting that HTLV‐1 cfDNA may potentially be used as a surrogate biomarker for lymphomatous ATL, which may be a more superior method in this setting.

The histological features of lymph nodes in ATL patients are usually characterized by diffuse proliferation of atypical medium‐sized to large pleomorphic cells [18]. However, several morphological variants have been described that mimic angioimmunoblastic T‐cell lymphoma, anaplastic large‐cell lymphoma, and Hodgkin lymphoma. This morphological heterogeneity makes it difficult to distinguish ATL from other lymphomas, especially in patients with lymphoma type who present no abnormal lymphocytes in the peripheral blood. In such cases, measurement of HTLV‐1 cfDNA may be a prospective assay to increase the accuracy of the diagnosis of ATL.

In conclusion, quantification of HTLV‐1 cfDNA may serve as a promising biomarker for the identification of ATL development and its disease states in HTLV‐1‐infected individuals. We assessed the utility of HTLV‐1 cfDNA in a limited number of patients, especially in acute and lymphoma types, and have not yet evaluated an association between HTLV‐1 cfDNA amount and the prognoses of ATL patients. These early data warrant a prospective large‐cohort study to further validate the utility of quantification of HTLV‐1 cfDNA as a predictive factor for ATL development and minimal residual disease detection after treatment.

## AUTHOR CONTRIBUTIONS

Hiroo Katsuya conceptualized the study, collected and analyzed data, and prepared the manuscript. Hideaki Nakamura and Aya Maeda performed the experiment. Keitaro Ishii, Toshiaki Nagaie, Haruhiko Sano, Haruna Sano, Hidekazu Itamura, Sho Okamoto, Toshihiko Ando, Toshiki Watanabe, Kaoru Uchimaru, and Eisaburo Sueoka provided clinical samples and data. Yorifumi Satou and Shinya Kimura conceptualized the study, and reviewed the results and manuscript. All authors have approved the submitted final version of the manuscript.

## CONFLICT OF INTEREST STATEMENT

The authors declare they have no conflicts of interest.

## ETHICS STATEMENT

This study was approved by the existing institutional review board at Saga University.

## PATIENT CONSENT STATEMENT

All patients gave written informed consent in accordance with the Declaration of Helsinki.

## PERMISSION TO REPRODUCE MATERIAL FROM OTHER SOURCES

We permit to reproduce material from other sources.

## Supporting information

Supporting Information

## Data Availability

The data that support the findings of this study are available in the supporting material of this article.
