# Supplementary material for: HTLV‐1 cell‐free DNA in plasma as a potential biomarker in HTLV‐1 carriers and adult T‐cell leukemia‐lymphoma
Source: EJHaem. 2023 May 26;4(3):733–7. doi: 10.1002/jha2.725 (PMC10435724; doi:10.1002/jha2.725)
Supplement: Supplementary file 1 — Supporting Information [file JHA2-4-733-s001.docx]

| Supplementary table | | | | | | | | | | | |
| --- | --- | --- | --- | --- | --- | --- | --- | --- | --- | --- | --- |
| Dx | age | sex | PVL (%) | cf-HTLV1 DNA (copies/ml) | CADM1^+^ CD7^–^ in CD4^+^ cells | sIL-2R (U/ml) | Hb (g/dl) | WBC (cells/μl) | Ly (%) | abLy (%) | LDH (U/L) |
| AC | 66 | F | 5.76 | 9.8 | 1.3 | 478 | 14.0 | 5100 | 46.5 | 0.0 | . |
| AC | 47 | F | 1.15 | 1.4 | 1.2 | 284 | 7.7 | 4700 | 45.0 | 1.0 | 183 |
| AC | 61 | F | 20.09 | 17.5 | 36.5 | 632 | 13.2 | 7300 | 32.9 | 1.5 | 146 |
| AC | 68 | M | 4.50 | 0.0 | 6.5 | 611 | 15.6 | 4900 | 29.5 | 0.5 | 182 |
| AC | 68 | F | 5.57 | 0.0 |  | 296 | 12.5 | 5000 | 23.5 | 1.0 | . |
| AC | 73 | F | 9.68 | 4.4 | 6.3 | 568 | 12.3 | 6800 | 28.5 | 0.5 | . |
| AC | 73 | F | 14.56 | 3.8 | 15.5 | 263 | 13.6 | 5700 | 35.0 | 1.0 | 172 |
| AC | 78 | M | 2.52 | 0.0 | 3.3 | 781 | 11.8 | 5100 | 20.5 | 0.5 | 227 |
| AC | 65 | F | 0.10 | 1.4 | 0.3 | 150 | 12.4 | 3800 | 33.0 | 0.0 | . |
| AC | 64 | F | 10.08 | 0.0 | 8.0 | 311 | 13.6 | 4900 | 42.0 | 0.5 | 190 |
| AC | 39 | F | 10.71 | 33.8 | 7.9 | 303 | 13.2 | 4300 | 38.5 | 0.0 | . |
| AC | 36 | F | 0.90 | 0.0 | 1.4 | 339 | 12.4 | 10400 | 19.5 | 0.0 | . |
| AC | 63 | M | 0.13 | 0.0 | 0.3 | . | 15.3 | 10800 | 24.5 | 0.5 | . |
| AC | 40 | F | 10.10 | 13.0 | 2.7 | 373 | 13.1 | 7300 | 28.0 | 1.5 |  |
| AC | 80 | F | 2.05 | 0.0 | 0.9 | 405 | 15.2 | 10500 | 28.0 | 0.0 | . |
| AC | 31 | F | 5.44 | 0.0 |  | 360 | 11.2 | 6000 | 29.0 | 0.5 | . |
| AC | 58 | F | 10.96 | 12.5 |  | . | 15.2 | 6700 | 31.5 | 0.5 | 181 |
| AC | 55 | F | 20.93 | 8.8 | 11.4 | 463 | 12.8 | 7500 | 29.0 | 3.0 | 214 |
| AC | 85 | M | 1.45 | 0.0 | 11.8 | 457 | 11.7 | 4500 | 19.5 | 1.0 | . |
| AC | 49 | F | 0.30 | 0.0 | 0.1 | 243 | 14.1 | 4400 | 46.0 | 0.0 | 154 |
| AC | 52 | M | 18.09 | 1.4 | 15.0 | 370 | 15.0 | 4300 | 37.5 | 3.5 | 188 |
| AC | 67 | F | 6.15 | 0.0 | 5.1 | 277 | 14.2 | 5800 | 45.0 | 0.5 | 226 |
| AC | 70 | F | 8.44 | 0.0 | 8.0 | 640 | 15.5 | 5000 | 10.0 | 1.0 | 198 |
| AC | 41 | F | 6.69 | 13.8 | 4.3 | 290 | 12.4 | 5300 | 26.5 | 0.5 | . |
| AC | 43 | F | 8.76 | 23.8 | 7.4 | 401 | 13.5 | 6100 | 36.0 | 4.0 | 150 |
| AC | 58 | M | 1.22 | 8.8 | 2.3 | 350 | 13.4 | 6100 | 22.5 | 0.0 | . |
| AC | 31 | M | 0.24 | 0.0 | 0.5 | 184 | 14.9 | 9500 | 21.0 | 0.0 |  |
| AC | 55 | F | 7.53 | 0.0 | 8.6 | 440 | 13.6 | 5300 | 24.0 | 0.0 | . |
| AC | 74 | F | 9.19 | 7.5 | 10.1 | 298 | 12.0 | 6200 | 19.0 | 0.5 | 175 |
| AC | 77 | M | 11.15 | 8.8 | 18.0 | 392 | 14.2 | 8900 | 34.0 | 1.5 | 179 |
| AC | 40 | F | 13.70 | 16.3 | 11.8 | 392 | 11.5 | 5400 | 23.0 | 1.0 | 149 |
| AC | 47 | M | 16.00 | 1.4 | 19 | 483 | 13.9 | 4100 | 26.5 | 1.5 | . |
| AC | 66 | M | 8.45 | 15.0 | 9.2 | 719 | 16.5 | 12800 | 37.0 | 1.5 | 114 |
| AC | 79 | F | 9.84 | 0.0 | 14.0 | 253 | 12.7 | 4500 | 31.0 | 2.0 | 188 |
| AC | 64 | F | 2.35 | 0.0 | 0.7 | 296 | 13.9 | 9500 | 14.0 | 0.0 | 241 |
| AC | 46 | F | 9.44 | 0.0 | 15.8 | 353 | 13.1 | 4500 | 12.0 | 0.5 | . |
| AC | 61 | F | 3.28 | 4.2 | 5.2 | 574 | 14.0 | 5000 | 33.0 | 1.0 | 220 |
| AC | 48 | F | 5.72 | 0.0 | 5.2 | 245 | 13.4 | 4800 | 28.0 | 1.5 | 139 |
| AC | 45 | F | 5.34 | 8.8 | 3.6 | 242 | 10.8 | 4800 | 21.0 | 1.0 | . |
| AC | 65 | M | 1.31 | 0.0 | 4.5 | 340 | 15.6 | 6800 | 30.5 | 1.0 | 157 |
| AC | 82 | M | 0.16 | 0.0 | 1.9 | 3545 | 10.9 | 3000 | 15.0 | 0.0 | 920 |
| AC | 78 | F | 0.0 | 0.0 |  | 10514 | 9.4 | 10600 | 3.5 | 0.0 | 2273 |
| acute | 58 | F | 84.04 | 200.0 | 77.6 | 6214 | 12.4 | 11800 | 33.0 | 38.0 | 263 |
| acute | 76 | F | 54.67 | 1550.0 | 82.5 | 11479 | 12.6 | 6300 | 12.0 | 39.0 | 331 |
| acute | 74 | F | 9.14 | 68.8 | 36.2 | 80270 | 15.3 | 13500 | 2.5 | 2.0 | 703 |
| acute | 61 | F | 88.53 | 1050.0 | 98.4 | 71686 | 11.6 | 25500 | 16.5 | 73.5 | 891 |
| chronic | 72 | F | 81.74 | 30.0 | 91.9 | 3032 | 13.3 | 11900 | 40.5 | 24.0 | 268 |
| chronic | 62 | M | 74.44 | 108.8 | 39.2 | 2210 | 15.3 | 11200 | 20.5 | 41.5 | 180 |
| chronic | 86 | F | 67.14 | 50.0 | 38.1 | 1271 | 13.5 | 13500 | 31.5 | 18.5 | 209 |
| chronic | 63 | F | 212.36 | 56.3 | 91.1 | 4632 | 13.0 | 12700 | 14.0 | 46.0 | 168 |
| chronic | 79 | F | 75.97 | 1212.5 | 1.1 | 9401 | 11.1 | 10800 | 4.5 | 47.0 | 191 |
| chronic | 72 | M | 14.48 | 28.8 | 29.6 | 1097 | 14.1 | 8600 | 42.4 | 1.5 | 172 |
| chronic | 66 | F | 85.59 | 237.5 | 83.4 | 19095 | 12.7 | 33900 | 4.0 | 79.0 | 378 |
| lymphoma | 76 | M | 1.17 | 1087.5 | 4.7 | 76200 | 12.2 | 4300 | 29.0 | 0.0 | 401 |
| lymphoma | 82 | F | 14.75 | 1650.0 | 34.6 | 135600 | 10.6 | 7100 | 9.5 | 0.7 | 593 |
| lymphoma | 72 | F | 8.47 | 3787.5 | 6.5 | 185600 | 11.5 | 5800 | 14.5 | 1.0 | 303 |
| lymphoma | 61 | M | 9.96 | 2812.5 | 31.6 | 27239 | 11.3 | 11400 | 1.0 | 0.0 | 819 |
| lymphoma | 71 | M | 5.43 | 837.5 | 6.8 | 6155 | 13.9 | 6900 | 14.0 | 0.0 | 249 |
| smoldering | 70 | F | 19.80 | 17.5 | 27.6 | 474 | 11.4 | 4000 | 39.0 | 7.0 | 155 |
| smoldering | 45 | F | 13.68 | 47.5 | 9.8 | . | 12.3 | 6700 | 32.0 | 2.0 | 214 |
| smoldering | 77 | F | 10.14 | 287.5 | 15.5 | 748 | 13.2 | 5400 | 25.0 | 2.5 | 242 |
| smoldering | 44 | M | 16.33 | 8.8 | 18.5 | 523 | 15.1 | 3100 | 22.5 | 2.0 | 183 |
| smoldering | 79 | M | 60.12 | 112.5 | 83.6 | 1008 | 2.3 | 6700 | 15.5 | 25.5 | 151 |
| smoldering | 73 | M | 1.02 | 8.8 | 4.5 | 1501 | 13.5 | 7500 | 20.0 | 0.0 | 216 |
| smoldering | 63 | F | 24.68 | 8.8 | 27.7 | 499 | 13.7 | 3900 | 43.5 | 4.5 | 228 |
| smoldering | 66 | M | 58.13 | 83.8 | 28.9 | 2265 | 13.7 | 7500 | 28.0 | 19.0 | 192 |
| smoldering | 72 | F | 0.00 | 0.0 | 5.6 | 964 | 10.7 | 6100 | 21.0 | 14.0 | 209 |
